# A Multicenter, Randomized, Double-Blind, Placebo-Controlled Trial of High-Dose Rebamipide Treatment for Low-Dose Aspirin-Induced Moderate-to-Severe Small Intestinal Damage

**DOI:** 10.1371/journal.pone.0122330

**Published:** 2015-04-15

**Authors:** Toshio Watanabe, Toshihisa Takeuchi, Osamu Handa, Yasuhisa Sakata, Tetsuya Tanigawa, Masatsugu Shiba, Yuji Naito, Kazuhide Higuchi, Kazuma Fujimoto, Toshikazu Yoshikawa, Tetsuo Arakawa

**Affiliations:** 1 Department of Gastroenterology, Osaka City University Graduate School of Medicine, Osaka, Japan; 2 Second Department of Internal Medicine, Osaka Medical College, Takatsuki, Osaka, Japan; 3 Department of Molecular Gastroenterology and Hepatology, Kyoto Prefectural University of Medicine, Kyoto, Japan; 4 Department of Internal Medicine and Gastroenterology, Saga Medical School, Saga, Japan; University Hospital Llandough, UNITED KINGDOM

## Abstract

**Background:**

Low-dose aspirin (LDA) frequently causes small bowel injury. While some drugs have been reported to be effective in treating LDA-induced small intestinal damage, most studies did not exclude patients with mild damage thought to be clinically insignificant.

**Aim:**

We conducted a multicenter, randomized, double-blind, placebo-controlled trial to assess the efficacy of a high dose of rebamipide, a gastroprotective drug, for LDA-induced moderate-to-severe enteropathy.

**Methods:**

We enrolled patients who received 100 mg of enteric-coated aspirin daily for more than 3 months and were found to have more than 3 mucosal breaks (i.e., erosions or ulcers) in the small intestine by capsule endoscopy. Eligible patients were assigned to receive either rebamipide 300 mg (triple dose) 3 times daily or placebo for 8 weeks in a 2:1 ratio. Capsule endoscopy was then repeated. The primary endpoint was the change in the number of mucosal breaks from baseline to 8 weeks. Secondary endpoints included the complete healing of mucosal breaks at 8 weeks and the change in Lewis score (an endoscopic score assessing damage severity) from baseline to 8 weeks.

**Results:**

The study was completed by 38 patients (rebamipide group: n = 25, placebo group: n = 13). After 8 weeks of treatment, rebamipide, but not placebo, significantly decreased the number of mucosal breaks (p = 0.046). While the difference was not significant (p = 0.13), the rate of complete mucosal break healing in the rebamipide group (32%, 8 of 25) tended to be higher than that in the placebo group (7.7%, 1 of 13). Rebamipide treatment significantly improved intestinal damage severity as assessed by the Lewis score (p = 0.02), whereas placebo did not. The triple dose of rebamipide was well tolerated.

**Conclusions:**

High-dose rebamipide is effective for the treatment of LDA-induced moderate-to-severe enteropathy.

**Trial Registration:**

UMIN Clinical Trials Registry UMIN000003463

## Introduction

While it is prescribed widely for the primary or secondary prevention of cardiovascular or cerebrovascular disease [1–3], low-dose aspirin (LDA) increases the risk of gastrointestinal ulceration and bleeding. In the last decade, concerns over this adverse effect of LDA focused mainly on the upper gastrointestinal tract. However, several studies used video capsule endoscopy (VCE) to show that LDA frequently causes injury to small bowel [4, 5], inducing intestinal bleeding and anemia [6]. Thus, LDA-induced enteropathy has recently been recognized as clinically significant.

Several clinical trials were performed with the aim of overcoming this adverse effect of LDA on the small intestine, and some drugs were shown to effectively heal LDA-induced small intestinal damage [4, 7, 8]. However, these studies did not exclude patients with mild damage thought to be clinically insignificant. Furthermore, few randomized double-blind trials supporting the efficacy of treatments for LDA-induced enteropathy have been reported.

Rebamipide (2-(4-chlorobenzoylamino-3-[2(1H)-quinolinon-4-yl] propionic acid)), a gastroprotective drug, has been clinically proven to effectively heal gastric ulcers [9] and prevent non-steroidal anti-inflammatory drug (NSAID)-induced gastroduodenal damage [10]. Recent studies using VCE have shown that rebamipide also has anti-ulcer effects in the small intestine, preventing both LDA- and NSAID-induced enteropathy in healthy volunteers [11, 12]. This suggests that rebamipide may also be effective in treating LDA-induced enteropathy. Importantly, Akamatsu et al. reported that rebamipide exhibited anti-ulcer effects via direct local penetration from inside the stomach to the gastric mucosa, but not via systemic delivery after absorption in the gastrointestinal tract [13]. Therefore, the ulcer-healing effect of rebamipide on the small intestine appears weak at the standard dose of 300 mg/day, so a higher dose is thought to be needed to successfully treat small intestine damage, especially LDA-induced severe enteropathy. Although the dose of rebamipide currently approved for gastric ulcer and gastritis is 300 mg/day, doses up to 900 mg/day were investigated in a phase II dose-finding study conducted in Japan, and no safety problems were noted. Hence, we conducted a multicenter, randomized, double-blind, placebo-controlled trial to assess the efficacy of triple-dose rebamipide in the treatment of LDA-induced moderate-to-severe small intestinal damage.

## Methods

The protocol of this trial and the supporting CONSORT checklist are available as supporting information; see S1 CONSORT Checklist and S1 Protocol.

### Ethical approval

Before the start of the study, the Institutional Review Board of each participating institution reviewed and approved the protocol: The ethics committee of the Osaka City University Graduate School of Medicine (approval number, 1619); The ethics committee of the Osaka Medical College (approval number, 705): The ethics committee of the Kyoto Prefectural University of Medicine (approval number, C-633); and The ethics committee of the Saga Medical School (approval number, 2012-10-01). Written informed consent was obtained from all participants

### Study design

This Cytoprotection by REbamipide on Aspirin-induced Mid-GI damage (CREAM) study was a multicenter, randomized, double-blind, placebo-controlled trial conducted to assess the efficacy and safety of triple-dose rebamipide for LDA-induced moderate-to-severe small intestinal damage. The study was carried out at 4 medical centers (Osaka City University Hospital, Osaka Medical College Hospital, Kyoto Prefectural Medical University Hospital, and Saga University Hospital) in Japan from February 2011 through January 2014. The study was conducted according to the Declaration of Helsinki and Good Clinical Practice guidelines. This trial was registered in the UMIN Clinical Trials Registry as UMIN000003463.

Eligible patients were assigned to receive either rebamipide 300 mg 3 times daily or placebo for 8 weeks in a 2:1 allocation ratio. Randomization was achieved by the minimization method, and stratified by site of enrollment and use of concomitant antiplatelet drugs. After 8 weeks of treatment with rebamipide or placebo, VCE was performed to assess the impact of treatment on small intestinal damage.

### VCE procedure

VCE was performed using the PillCam SB2 (Given Imaging, Ltd, Yoqneam, Israel). Patients fasted for 12 h before swallowing the capsule. Fluids were permitted 2 h later, followed by a light meal another 2 h later. Data were collected for up to 8 h after capsule ingestion. After 8 h, the sensor array and recording device were removed. The VCE digital image stream was then reviewed by 4 experts (Tetsuya T, Toshihisa T, OH, and YS). Since it is difficult to definitively distinguish between erosions and ulcers (because VCE cannot accurately assess either lesion size or depth), we combined them into a single category of injury (mucosal breaks).

In addition, the severity of LDA-induced enteropathy was assessed with the Lewis score (LS) [14], a capsule endoscopic grading system of small intestinal mucosal inflammation and damage. The detailed methods of calculating the LS have been previously described [14]. Small bowel inflammatory changes were categorized into 3 groups according to the LS: normal or clinically insignificant change (LS <135), mild mucosal inflammatory change (LS 135–790), and moderate or severe change (LS >790).

### Patients

Male or female outpatients were eligible for the trial if they received 100 mg of enteric-coated aspirin daily for more than 3 months for the primary or secondary prevention of cardiovascular and cerebrovascular disease and were found to have more than 3 mucosal breaks (i.e., erosions or ulcers) in the small intestine by capsule endoscopy. Other inclusion criteria were an age of at least 20 years and stable disease with no pressing need to change the current aspirin regimen.

The main exclusion criteria included diseases that could induce small intestinal injury (such as Crohn’s disease, gastrointestinal stromal tumors, and small intestinal tumors), use of NSAIDs or anti-cancer drugs, the presence of diverticula and diaphragm-like stenosis in the small intestine, and severe renal or hepatic dysfunction. Patients were also excluded if they used antibiotics, sulfasalazine, prostaglandin (PG) analogs, corticosteroids, or gastroprotective drugs, as these could potentially heal or worsen LDA-induced small intestinal damage. Patients with ongoing overt gastrointestinal bleeding and those found to have active small intestinal bleeding by VCE were also excluded.

### Assessment

The primary endpoint was the change in the number of mucosal breaks from baseline to 8 weeks. Secondary endpoints included changes in the LS from baseline to 8 weeks, complete healing of small intestinal mucosal breaks at 8 weeks, and changes in the levels of hemoglobin and serum albumin from baseline to 8 weeks. Safety assessments included the documentation of adverse events and clinical laboratory testing at baseline and after 4 and 8 weeks of treatment.

### Statistical analysis

Results are expressed as medians and interquartile ranges for continuous variables and percentages for categorical variables. The characteristics of patients in the placebo and rebamipide groups were compared using the χ2 test or Fisher exact test for categorical variables, whereas quantitative variables were analyzed by the Mann–Whitney *U*-test. The Wilcoxon signed-rank test (for continuous data) or McNemar test (for ordinal data) was used for comparisons of data between baseline and 8 weeks. Differences with P-values <0.05 were considered significant. All statistical analyses were performed using SPSS 21 for Windows (SPSS Inc.; Chicago, Illinois, United States).

### Sample size estimation

Sample size was estimated based on a previous study showing that the complete healing rate of LDA-induced mucosal breaks was 58% in patients who took misoprostol, a PGE_1_ analog, for 8 weeks. The following assumptions were made for sample size calculation: (1) the wound-healing effect of a triple dose of rebamipide was equal to that of misoprostol; (2) the complete healing rates in the placebo and rebamipide groups were 20% and 58%, respectively; (3) 10% of patients in each group will either drop out or have unsatisfactory follow-up VCE images. We calculated that at least 30 patients were needed in the rebamipide group to give the study 90% power at a 5% significance level by a test of proportions.

## Results

### Subject enrollment and baseline characteristics

**Fig 1:**
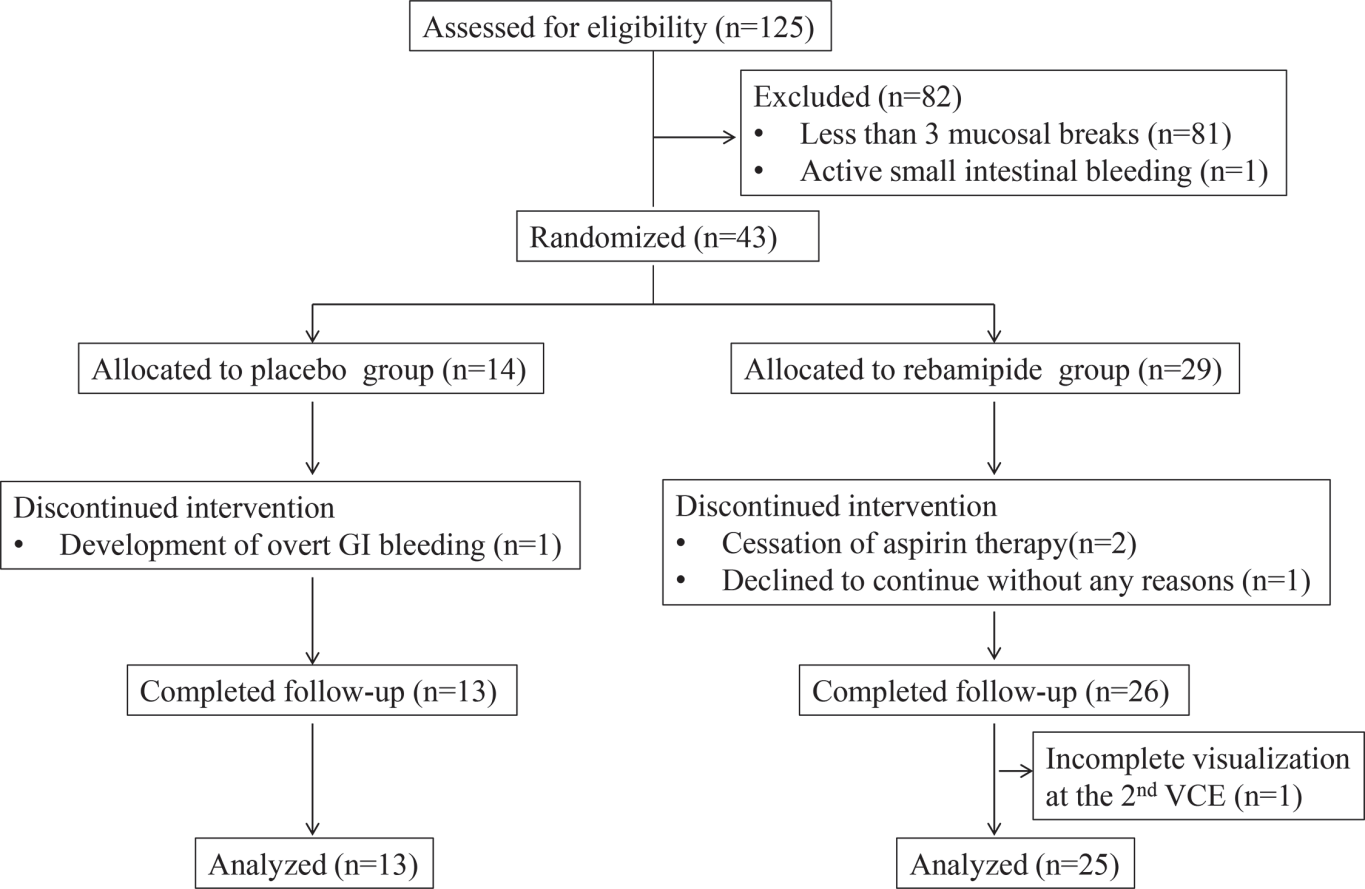
Trial flow diagram.

Between February 2011 and January 2014, 125 patients were assessed for study eligibility. In total, 43 patients were enrolled and randomly assigned (in a 2:1 ratio) to the rebamipide group (n = 29) or placebo group (n = 14). The study’s participant flow is shown in Fig 1. Five patients were excluded because of cessation of LDA therapy during the trial (n = 2), incomplete visualization on the second capsule endoscopy (n = 1), patient’s intention to withdraw from the trial (n = 1), and the development of overt gastrointestinal bleeding (n = 1).

Regarding gastrointestinal symptoms in the enrolled patients, one patient had abdominal fullness at baseline, while the others were asymptomatic. At 8 weeks of treatment, all patients who completed the trial were asymptomatic.

**Fig 2:**
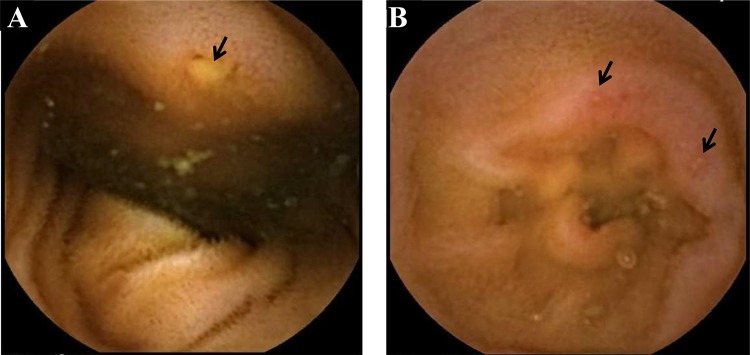
Capsule endoscopic images of small bowel mucosal breaks induced by low-dose enteric-coated aspirin. Typical photographs of mucosal breaks observed in this study are shown. A: a mucosal break (arrow). B: multiple mucosal breaks (arrows).

The patients’ baseline clinical characteristics were grouped according to treatment (Table 1). There was no significant difference in baseline characteristics such as age, sex, body mass index, smoking habit, and alcohol consumption between the placebo and rebamipide groups. In addition, the number of mucosal breaks at baseline did not differ between the two groups. Typical photographs of mucosal breaks observed in this study are shown in Fig 2A and 2B.

**Table 1:** Baseline characteristics of patients.

	Placebo (n = 13)	Rebamipide (n = 25)	*P* value
Median age (y)	74.8	74.1	0.93
Male sex, n (%)	9 (69.2)	16 (64.0)	1
Smoking habit, n (%)	1 (7.7)	4 (16)	0.64
Alcohol consumption, n (%)	4 (30.8)	10 (40)	0.73
Underlying diseases			
ischemic heart disease, n (%)	8 (32)	15 (60)	0.8
ischemic cerebrovascular disease, n (%)	3 (23.1)	3 (12)	0.39
atherosclerosis, n (%)	1 (7.7)	4 (16)	0.64
others, n (%)	1 (7.7)	3 (12)	1
Complications			
hypertension, n (%)	11 (84.6)	20 (80)	1
hyperlipidemia, n (%)	6 (46.2)	12 (48)	0.81
diabetes mellitus, n (%)	3 (27.3)	3 (12)	0.39
Proton pump inhibitor use, n (%)	7 (53.8)	12 (48)	1
H_2_ receptor antagonist use, n (%)	0 (0)	2 (8)	0.54
Concomitant use of other antiplatelets	2 (15.4)	3 (12)	0.11
Warfarin use, n (%)	1 (7.7)	3 (12)	1
ARB use, n (%)	7 (53.8)	12 (48)	1
Statin use, n (%)	6 (46.2)	12 (48)	0.81
Median number of mucosal breaks (IQR)	6 (4.0–18.5)	4 (3.0–8.0)	0.57
Median hemoglobin level (IQR) (g/dL)	13.1 (9.6–14.1)	12.9 (11.3–13.6)	1
Median albumin level (IQR) (g/dL)	4 (3.8–4.3)	4.2 (3.9–4.4)	0.78

ARB, angiotensin II receptor blocker; IQR, interquartile range.

### Primary endpoint

**Fig 3:**
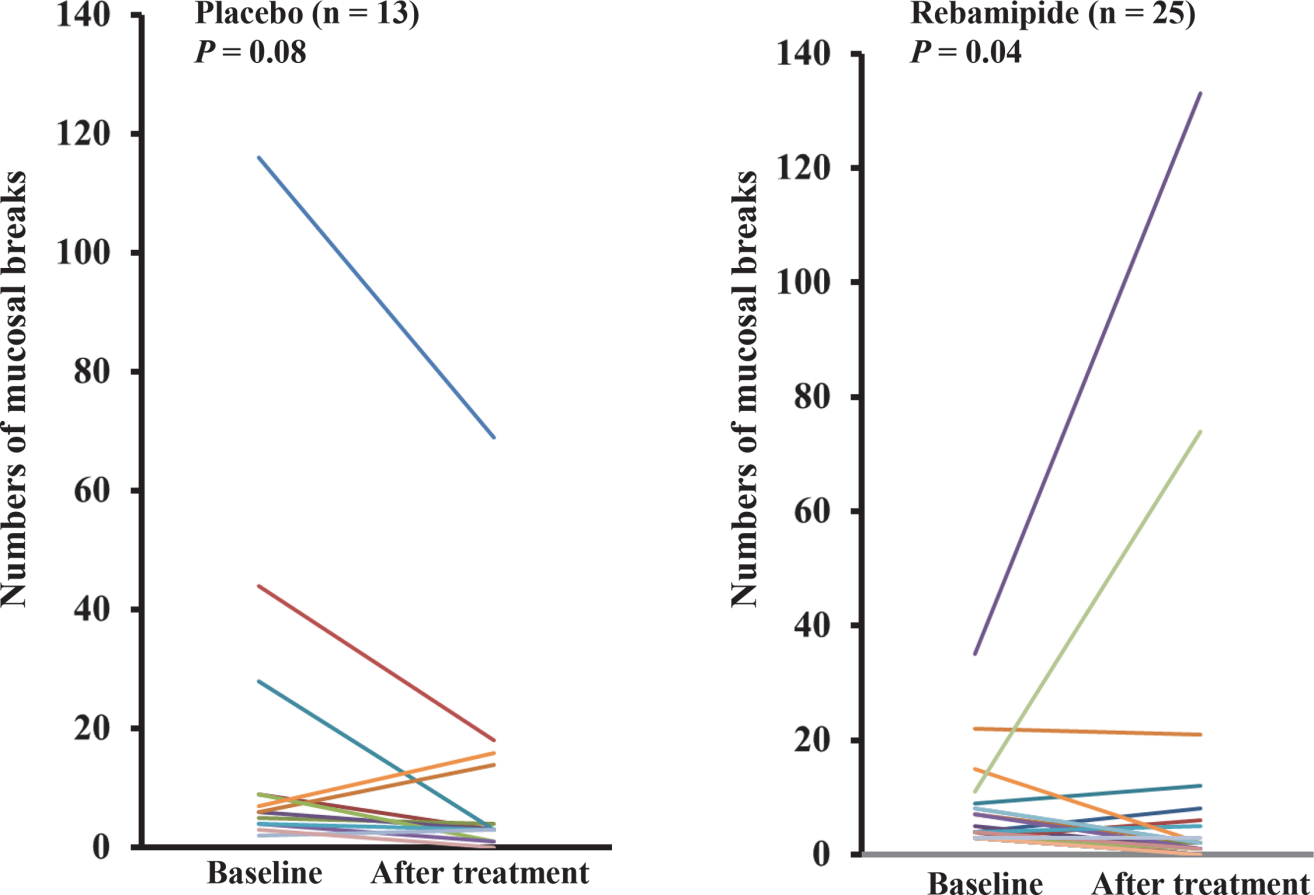
Changes in the number of small intestinal mucosal breaks from baseline to posttreatment. Results were analyzed by the Wilcoxon signed-rank test.

After 8 weeks of treatment, rebamipide significantly reduced the number of mucosal breaks from 4.0 (3.0–8.0) to 2.0 (3.0–8.0, p = 0.046), whereas placebo did not result in a significant reduction (p = 0.08). The median number of mucosal breaks in the placebo group was 6.0 (4.0–18.5) at baseline and 3.0 (2.0–15.0) at 8 weeks (Fig 3).

### Secondary endpoints

**Fig 4:**
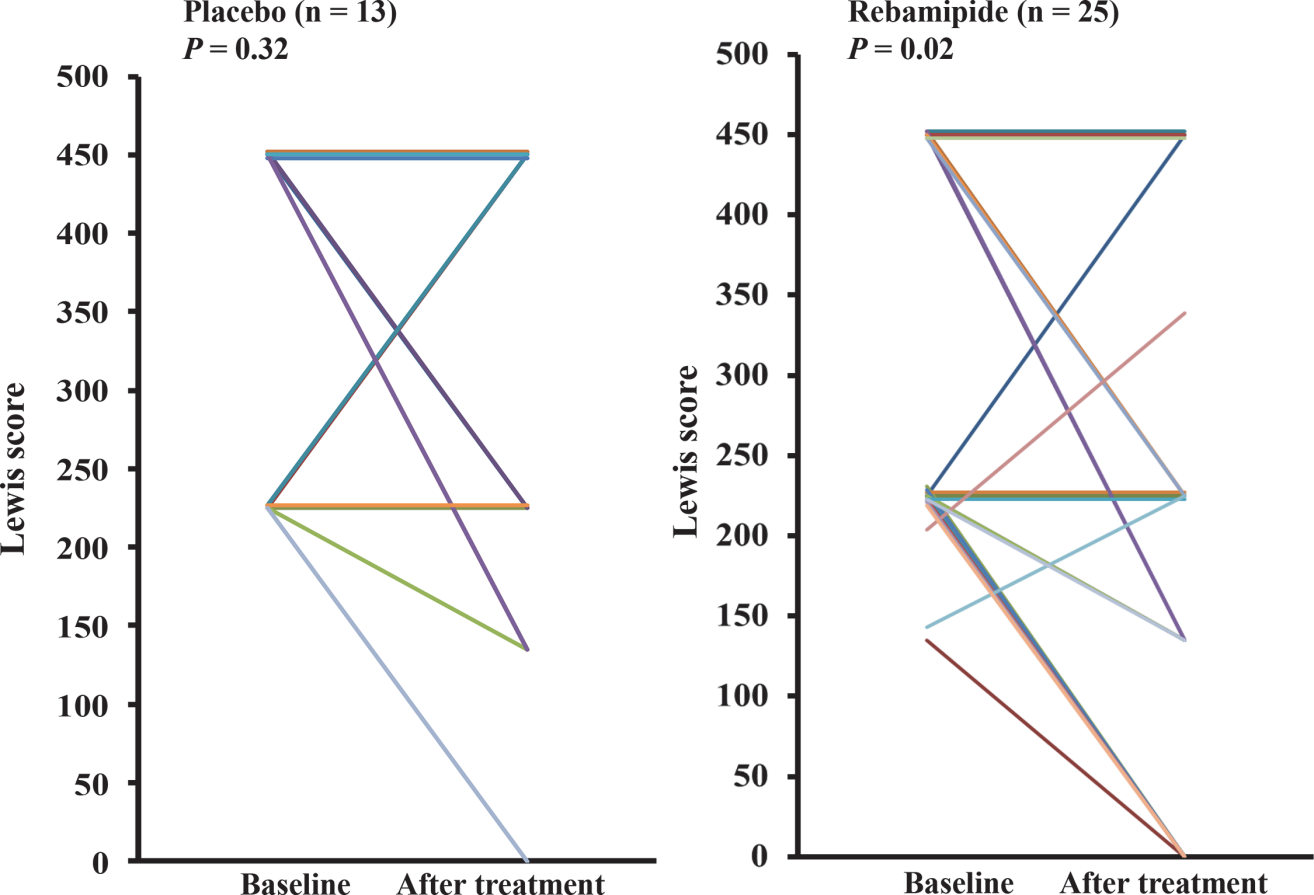
Changes in the severity of small intestinal mucosal breaks from baseline to posttreatment. Results were analyzed by McNemar test.

Rebamipide treatment significantly improved the severity of intestinal damage as assessed by the LS (p = 0.02), whereas placebo treatment did not alter the LS (p = 0.32, Fig 4). Eight (32%) of 25 patients in the rebamipide group achieved complete healing of the intestinal mucosal breaks at 8 weeks of treatment, compared with 1 (7.7%) of 13 patients in the placebo group. The complete healing rate in the rebamipide group was higher than that in the placebo group, although the difference did not reach statistical significance (p = 0.13). Neither group showed significant changes from baseline to 8 weeks in hemoglobin and serum albumin levels (Table 2).

**Table 2:** Changes in the levels of hemoglobin and albumin after treatment with placebo or rebamipide.

	Baseline	After treatment	*P* value	
Median hemoglobin level (IQR) (g/dL)				
Placebo	13.1 (9.6–14.1)	13.1 (11.0–14.1)		0.62
Rebamipide	12.9 (11.3–13.6)	13.2 (11.8–14.1)		0.19
Median albumin level (IQR) (g/dL)				
Placebo	4.0 (3.8–4.3)	4.1(3.9–4.2)		0.14
Rebamipide	4.2 (3.9–4.4)	4.2 (4.1–4.3)		0.77

Results are expressed as medians and interquartile ranges (IQRs)

### Safety

Significant changes from the baseline values of any of the examined laboratory parameters were recorded in both groups of patients. As described above, one patient in the placebo group developed overt gastrointestinal bleeding and discontinued the intervention (Fig 1); no other adverse events were reported.

## Discussion

Recent studies demonstrated the efficacy of several drugs such as rebamipide [12], misoprostol [4] and a probiotic preparation, *Lactobacillus casei* strain Shirota [7], for LDA-induced enteropathy. However, these studies had shortcomings in terms of the subjects included. Some studies were conducted in young, healthy volunteers and evaluated preventive effects on LDA-induced small intestinal damage. Even those studies that evaluated healing effects in chronic LDA users enrolled patients with red spots but no mucosal breaks or patients with very few mucosal breaks thought to be clinically insignificant. Furthermore, few randomized, double-blind, placebo-controlled trials assessing the efficacy of treatments for LDA-induced intestinal damage have been reported. In this study, we demonstrated that treatment with triple-dose rebamipide could be effective therapy for LDA-induced enteropathy in patients who had more than 3 mucosal breaks, and that this dose of rebamipide was well tolerated and did not cause any adverse events. To the best of our knowledge, rebamipide is the first drug to show efficacy for the treatment of LDA-induced moderate-to-severe enteropathy.

Recent studies greatly improved our understanding of the mechanisms underlying the pathogenesis of NSAID (including LDA)-induced small bowel damage. A mechanism of primary importance for the onset of this damage is the inhibition of cyclooxygenase by NSAIDs, which leads to collapse of the mucosal defensive system due to a reduction in PG synthesis. In addition, the topical effect of NSAIDs on the small bowel epithelium causes mitochondrial dysfunction, which also contributes to the disturbance of barrier function [15]. Once the mucosal barrier has been disrupted, luminal Gram-negative bacteria enter the epithelium and release lipopolysaccharide [16], resulting in the induction of mucosal inflammatory responses such as cytokine overexpression via the activation of the innate immune system [16–19]. This phenomenon then triggers neutrophil infiltration with subsequent release of tissue proteolytic enzymes and reactive oxygen species, leading to intestinal ulceration.

Therefore, agents that interfere with any phase of the development of the damage are thought to be useful in treating enteropathy [4, 20, 21]. Rebamipide has various effects on the gastrointestinal tract [9, 22], which explains its efficacy against LDA- or NSAID-induced small intestinal damage. It increases PG concentration via the induction of cyclooxygenase-2 [23, 24] and has the potential to inhibit inflammatory cytokine expression [25] and neutrophil infiltration [26]. Rebamipide can also scavenge reactive oxygen species [27] and prevent indomethacin-induced mitochondrial damage in cultured gastric cells [28]. Furthermore, recent experimental studies showed that rebamipide regulates small intestinal microbiota via the induction of antimicrobial peptides [29, 30]. Thus, these effects of rebamipide appear to act in concert to prevent and heal enteropathy.

There are a few limitations to this study. First, the sample size is relatively small. We therefore failed to find a significant difference in the complete healing rate of mucosal breaks between the rebamipide and placebo groups, although we successfully demonstrated the efficacy of rebamipide in terms of the primary endpoint (i.e., reduction in the number of mucosal breaks). The sample size of this trial was calculated based on our previous study in which treatment with misoprostol resulted in complete healing in 58% of patients with LDA-induced small intestinal mucosal breaks [4]. However, that study did not exclude patients with 1 or 2 mucosal breaks. As mentioned previously, we excluded such patients and enrolled only patients with more than 3 mucosal breaks, which may have reduced the complete healing rate in this study. Further studies involving larger sample sizes are needed to determine the efficacy of rebamipide for achieving complete healing of moderate-to-severe enteropathy. The second limitation is our exclusion of patients with active small intestinal bleeding, which is a clinically important condition. Therefore, a clinical trial that includes such patients should be performed next. The other limitation is that we cannot deny the possibility that VCE missed some intestinal lesions because the LDA-induced small intestinal mucosal breaks were relatively small. A large-scale trial would be required to decrease the effect of this limitation.

In conclusion, triple-dose rebamipide is effective in the treatment of LDA-induced moderate-to-severe enteropathy, and is well tolerated and safe. Since our study does not repudiate the efficacy of standard dose rebamipide, a comparative study of standard and triple-dose rebamipide is needed to determine the appropriate dose of this drug for the treatment of LDA-induced clinically significant small intestinal damage.

## Supporting Information

S1 CONSORT Checklist CONSORT Checklist. (DOC)

S1 Protocol Trial Protocol. (DOC)
