# Supplementary material for: A Multicenter, Randomized, Double-Blind, Placebo-Controlled Trial of High-Dose Rebamipide Treatment for Low-Dose Aspirin-Induced Moderate-to-Severe Small Intestinal Damage
Source: PLoS One. 2015 Apr 15;10(4):e0122330. doi: 10.1371/journal.pone.0122330 (PMC4398323; doi:10.1371/journal.pone.0122330)
Supplement: S1 Protocol — (DOC) [file pone.0122330.s002.doc]

**Clinical Study Protocol**

A multicenter, randomized, double-blind, placebo-controlled trial of high-dose rebamipide treatment for low-dose aspirin-induced moderate-to-severe small intestinal damage (CREAM study)

1. Introduction

Aspirin has been used for defervescence and analgesia for a long time; however, in recent years, low-dose preparations with an expected antiplatelet effect have frequently been used. This effect is based on the inhibition of prostaglandin or thromboxane A2 production by the inhibition of cyclooxygenase activity.^1^ In 2002, the American Heart Association recommended the prophylactic administration of low-dose aspirin (LDA) for the recurrence of ischemic heart disease. This recommendation was based on the finding that LDA inhibited the recurrence of heart disease by 10% during a 10-year observation period.^2^ Owing to the effect of the recommendation, prophylactic administration of LDA at a dose of 100 mg/day or lower for ischemic heart disease became widely used.

However, despite such clinical contribution, gastrointestinal adverse reactions arose as problems.^3-5^ A recent case-control study in Japan reported that the administration of LDA increases the risk of upper gastrointestinal bleeding by 5.5-fold compared to that with nontreatment with aspirin.^6^ To solve this problem, prophylactic administration of antisecretory drugs is recommended worldwide, and clinical trials to obtain the indication are under way in Japan.

Meanwhile, another problem that arose is the presence of cases in which gastrointestinal bleeding other than gastric, duodenal, and large intestinal bleeding is suspected, and the number of patients in 1999 was reported to be as many as 45,000 based on the estimation of the incidence of peptic ulcer (bleeding) of unknown site performed by Japanese the Ministry of Health, Labour and Welfare once every 3 years. Endoscopy through the oral and transanal routes has generally been performed; however, diagnosis in the small intestine, a long organ with a length of approximately 7 m, has been inadequate. Recently, the development of capsule endoscopy and balloon endoscopy has allowed the observation of the entire small intestine, enabling the detection of small intestinal diseases such as small intestinal bleeding and small intestinal tumors.^7-9^ In Spain, an epidemiologic study in >30,000 subjects has been conducted, and death due to lower gastrointestinal disease was reported in 8.8% in 2005, which was a significant increase compared with the 5.5% rate in 2001. A recent increase in patients taking LDA has been pointed out as one of the causes.^10^ This is considered to be because of the difficulty of antisecretory drugs to act on the small intestine.

In our previous study, a significant improvement effect was observed after an 8-week administration of misoprostol (prostaglandin preparation) for small intestinal damage (redness and mucosal break) in patients receiving long-term aspirin therapy.^11^ This finding suggests the possibility that prostaglandin preparations may act on the small intestine. However, because misoprostol is associated with a high incidence of adverse reactions, such as diarrhea, candidate drugs in addition to misoprostol need to be further investigated.

Therefore, we decided to perform an exploratory study by using the antiulcer drug rebamipide as a candidate drug. Rebamipide exerts a wound-healing effect independently of gastric acid, and is expected to be useful against small intestinal damage. Rebamipide has pharmacological effects such as endogenous prostaglandin-increasing, free radical–scavenging, microcirculation-improving, and anti-inflammatory effects, besides its prophylactic effect against nonsteroidal anti-inflammatory drug (NSAID)-induced small intestinal damage that has been reported in animal experiments.^12-13^ The dose of rebamipide currently approved for gastric ulcer and gastritis is 300 mg/day; however, a dose of 300 mg/day or higher is presumed to be necessary for the small intestine because of its large area. According to a clinical trial report on rebamipide, doses up to 900 mg/day have been investigated in a phase II dose-finding study, and no safety problems were noted.^14-15^ Therefore, in this exploratory study, we decided to investigate the efficacy of a dose of 900 mg.

**2. Study Objective**

To investigate the healing-promoting effect of rebamipide, a protective drug for LDA-induced small intestinal mucosal damage.

Because there is no evidence for the therapeutic effect of rebamipide against LDA-induced small intestinal damage, this study was created as a pilot study exploring this possibility.

**3. Study Method**

**3.1 Study Subjects**

Patients taking low-dose (enteric) aspirin (brand name, Bayaspirin) for 3 months or longer.

**3.1.1 Inclusion Criteria**

Subjects who meet the following conditions will be registered into this study:

1. Subjects who provided written consent for participation.
2. Subjects who have been receiving treatment with Bayaspirin (100 mg/day or lower) for at least 3 months, and who are scheduled to continue Bayaspirin for the study period of 8 weeks or longer.
3. Patients with diseases such as ischemic heart disease and ischemic cerebrovascular disease whose conditions are stable.
4. Subjects aged 20 years or older.
5. Subjects of both sexes.
6. Subjects with small intestinal damage detected with capsule endoscopy.

**3.1.2 Exclusion Criteria**

The following subjects will be excluded from the study:

1. Subjects with small intestinal lesions other than LDA-induced small intestinal damage, such as Crohn’s disease and small intestinal tumor.
2. Subjects taking drugs that may induce small intestinal damage such as NSAIDs and anticancer drugs.
3. Subjects taking drugs that may improve small intestinal lesions such as antibiotics, sulfasalazine, prostaglandin preparations, steroids, free radical inhibitors, elastase inhibitors, and gastric mucosa–protecting agents.
4. Subjects with overt bleeding from the small intestine.
5. Subjects with small intestinal diverticula and membranous stenosis.
6. Subjects with significantly decreased renal and hepatic function.
7. Subjects with a history of hypersensitivity to rebamipide.
8. Subjects who participated in other studies within 4 weeks before participation in this study.
9. Subjects judged to be inappropriate by the subinvestigator and the principal investigator.

**3.1.3 Handling of Patients**

1) The investigator will provide an adequate explanation about participation in this study, and will obtain consent from participants.

2) If some mucosal damage is observed on small intestinal endoscopy, the investigator will confirm that the subject satisfies the inclusion criteria and does not meet the exclusion criteria, and will register the subject into the study.

**3.1.4 Registration Method**

Central registration system

1. The investigator will fill out the registration communication form in accordance with the registration procedure and register the subject to the central registration center (see below) by fax.

Central registration center: Medical Division, Kondo Photo Process Co., Ltd.

11-15 Shimizudanicho, Tenoji-ku, Osaka

1. The central registration center will confirm whether the subject is eligible for this study.

• If the subject is eligible, a subject number—“(study site code), (registration number)”—will be assigned. The central registration center will specify that “the subject can be registered,” write the subject number in the registration confirmation form, and send the form to the investigator by fax.

• If the subject is not eligible, the central registration center will specify that “the subject cannot be registered,” write the reason in the registration confirmation form, and send the form to the investigator.

• The central registration center will retain all registration forms sent by fax.

1. The investigator will confirm the contents of the registration confirmation forms returned from the central registration center by fax.

• If the subject is registered, the fact that “the subject was registered” and the registration number will be specified in the subject screening list.

• If the subject is not registered, the fact that “the subject was not registered” will be specified in the subject screening list and the study will be completed at that time.

1. The investigator will retain all registration confirmation forms sent by fax.

**3.2 Study Design**

Classification based on the method of administration of the drug: A randomized, double-blind, placebo-controlled study.

Classification based on the contents of the study: A study to explore drug effect.

**3.3.1 Study Drug and Instrument Used**

Generic name: Rebamipide

Capsule endoscope (Given Image Diagnosis System manufactured by Given Imaging Ltd.)

**3.3.2 Test Drug**

(1) Generic name: Rebamipide

(2) Dosage form: White film-coated tablet

(3) Strength: One tablet = 100 mg rebamipide

(4) Storage: Room temperature

**3.3.3 Control Drug**

(1) Placebo

(2) Dosage form: Lactose

(3) Strength: One tablet = 100 mg lactose

(4) Storage: Room temperature

**3.3.4 Concomitant Drugs**

1) As described in the exclusion criteria in 3.1.3, drugs that affect the degree of small intestinal mucosal damage will be prohibited in principle during the study.

2) Drugs other than the above-mentioned prohibited concomitant drugs that have been used since before the initiation of administration of rebamipide can be concomitantly used as long as the dose is maintained constant during the study.

3) If patients with active gastric ulcer and duodenal ulcer are treated, an antisecretory drug (proton pump inhibitor, omeprazole) should be administered as necessary. However, mucosal protectants such as rebamipide should not be administered.

4) In patients taking mucosal protectants, the drugs should be discontinued by 2 weeks before capsule endoscopy.

5) If drugs other than the study drug are used, the drug name, dose, and administration period should be entered in the investigation form.

**3.3.5 Method and Period of Administration**

1) Patients with small intestinal damage among those taking Bayaspirin will be randomized into two groups (groups A and B). Capsule endoscopy will be used in the diagnosis of the small intestine. A separately specified controller will perform the allocation.

2) Randomization

Subjects will take the randomly assigned test drug during the study.

Dynamic allocation by using the minimization method will be applied in randomization, and subjects will be allocated to each of the two groups in the study. Drug numbers and information on the allocated groups will be managed by the allocation controller, and will be disclosed when the data are fixed after the completion of the study.

3) Allocated groups and drug administration method:

Group A: Three tablets per dose of control (placebo) tablet (100 mg lactose) will be taken three times daily after every meal.

Group B: Three tablets per dose of rebamipide tablet (100 mg rebamipide) will be taken three times daily after every meal.

4) Administration period: 8 weeks

**3.3.6 Contents and Schedule of Examination**

**Study design**

**Group A**

**(n=15)**

**Group B**

**(n=30)**

**Ｃａｐｓｕｌｅ　ｅｎｄｏｓｃｏｐｙ**

**pre**

**Ｂｌｏｏｄ　ｔｅｓｔ**

**Objective symptom**

**4 weeks**

**8 weeks**

**Ｓｕｂｊｅｃｔｉｖｅ　ｓｙｍｐｔｏｍ**

**3.3.6 Observation Items**

Subject background: Specified at registration

ID assigned by randomization, date of birth, sex, height, body weight, body mass index, drinking history (years), smoking history (years), underlying disease, complications, and used drugs (drug name, dose, and period of use)

Treatment compliance: Status of the administration of the drug

Hematology: Before administration, after administration (4 weeks), and at the completion of treatment (8 weeks)

Red blood cells (10^4^/μL), white blood cells (10^4^/μL), hemoglobin (g/dL), albumin (g/dL), hematocrit (%), total protein (g/dL), glutamic oxaloacetic transaminase (IU/L), glutamic pyruvic transaminase (IU/L), blood urea nitrogen (mg/dL), creatinine (g/day), iron (μg/dL), and C-reactive protein (μg/dL)

Subjective and objective symptoms: Before administration, after administration (4 weeks), and at the completion of treatment (8 weeks)

Subjective symptoms: Epigastric pain, abdominal distension, anorexia, heartburn, stomach heaviness, nausea, vomiting, belching, etc.

Objective symptoms: Epigastric tenderness, hematemesis, melena, etc.

Small intestinal endoscopy: Before administration and at the completion of treatment (8 weeks)

Stool frequency, abdominal symptoms, etc. (after examination; abdominal symptoms and capsule excretion symptoms will be confirmed)

**3.4 Evaluation Criteria**

**3.4.1 Primary Endpoint**

Decreasing the number of small intestinal mucosal breaks

**3.4.2 Secondary Endpoints**

1) Complete healing of small intestinal mucosal breaks

2) Improving the Lewis score

3) Improving hemoglobin and albumin levels

4) Safety evaluation

**3.5 Criteria for Study Termination and Dropout of Subjects**

1. Observation down to the cecum is not completed within the capsule endoscopy measurement time (8 h).
2. Adverse reactions judged to be serious, such as peptic ulcer, gastrointestinal bleeding, and perforation, develop.
3. Drugs described in the exclusion criteria are added.
4. The subject requests to discontinue study participation, and the investigator or principal investigator judges a discontinuation to be appropriate.
5. Compliance with treatment with the test drug during the study is <85%.

**3.6 Safety**

**3.6.1 Definition of Adverse Event**

Endoscopic findings, subjective symptoms, and objective findings for which clinically relevant changes meeting the following criteria are observed will be considered as adverse events.

1. Subjective symptoms and objective findings will be handled as adverse events if “development of a new abnormality” or “worsening” compared with baseline is observed in the investigation of subjective symptoms and objective findings after the completion of the study. The washout period will be included for subjective symptoms.
2. All cases of death will be handled as adverse events.
3. Adverse events for which a causal relation to the study drug cannot be clearly ruled out will be considered as adverse reactions.

**3.6.1.1 Actions against and handling of adverse events**

All adverse events revealed by endoscopy after administration, as well as subjective symptoms and objective findings will be investigated and recorded. The follow-up investigation of subjective symptoms and objective findings will be performed until symptoms become resolved. The follow-up period will be determined by the investigator.

**3.6.2.1 Handling of adverse reactions**

All adverse reactions revealed by endoscopy after administration, as well as the subjective symptoms and objective findings will be investigated and recorded. The follow-up investigation of subjective symptoms and objective findings will be performed until symptoms become resolved. The follow-up period will be determined by the investigator.

**3.6.3 Method of Evaluation of Adverse Events and Adverse Reactions**

- - - 1. **3.6.3.1 Investigation and recording of items**

1. Name of adverse event
2. Time and date of onset (detection)
3. Highest severity
4. Time and date of resolution
5. Time and date of outcome investigation and outcome (ongoing, resolving, or resolved)
6. Presence or absence, and details of treatment (necessity of treatment and the details of the newly performed treatment)
7. Judgment of a causal relation: The results of judgment of a causal relation between the adverse event and rebamipide will be entered in the investigation process. In particular, if the adverse event and rebamipide are judged to be “not related,” the reason should be clarified.
   - - 1. **3.6.3.2 Classification of the causal relation**

1: Not related

2: Probably not related

3: Definitely related

4: Unknown

**3.6.3.3 Definition of the classification of the causal relation**

1. Not related: A direct cause of the event other than the study drug can be clearly identified.

2. Probably related: The adverse event occurs and resolves with a proper temporal relation with the drug administration or blood (tissue and body fluid) concentration of the drug, and other factors are ruled out.

3. Definitely related: The adverse event occurs and resolves with a proper temporal relation with the drug administration or blood (tissue and body fluid) concentration of the drug, and any of the following is met:

• A similar finding is observed by readministration.

• The dose is confirmed to be toxic according to the measurement of drug concentration in the body.

• The drug sensitivity test shows a positive result on allergic symptoms.

4. Unknown: The above judgment cannot be made owing to the lack of information for the evaluation of a causal relation.

**3.6.3.4 Severity <<definition of severity>>**

1. Mild: The event does not particularly interfere with daily life.

2. Moderate: The event interferes with daily life.

3. Severe: The subject experiences great difficulty in daily living.

**3.6.4 Laboratory Test Values**

**3.6.4.1 Definition of laboratory abnormalities**

If clinically relevant changes meeting the following criteria are observed, laboratory test values will be considered as laboratory abnormalities:

1. Laboratory test values will be handled as laboratory abnormalities if “development of a new abnormality” or “worsening” compared with baseline is observed after the completion of the study.
2. Laboratory test values for which a causal relation to the study drug cannot be clearly ruled out will be considered as adverse reactions.

**3.6.4.2 Handling of laboratory abnormalities**

For all laboratory abnormalities, the following will be investigated and recorded. The follow-up investigation of laboratory abnormalities will be performed until normalization of the laboratory test values is confirmed. The follow-up period will be determined by the investigator. If drug withdrawal becomes necessary, symptoms that the subject notices during the withdrawal period and that may be harmful will be communicated to the investigator, as needed.

**3.7 Statistical Analysis**

**3.7.1 Analysis Method**

**3.7.1.1 Analysis of the subjects’ background before the initiation of the study**

Results are expressed as medians and interquartile ranges for continuous variables and percentages for categorical variables. The characteristics of patients in the placebo and rebamipide groups were compared using the χ_2_ test or Fisher exact test for categorical variables, whereas quantitative variables were analyzed by the Mann–Whitney U-test. The Wilcoxon signed-rank test (for continuous data) or McNemar test (for ordinal data) was used for comparisons of data between baseline and 8 weeks. Differences with P-values <0.05 were considered significant.

**3.7.1.2 Method of analysis of the primary and secondary endpoints**

The Wilcoxon signed-rank test (for continuous data) or McNemar test (for ordinal data) was used for comparisons of data between baseline and 8 weeks. Differences with P-values <0.05 were considered significant.

**3.7.1.4 Sample size**

Sample size was estimated based on a previous study showing that the complete healing rate of LDA-induced mucosal breaks was 58% in patients who took misoprostol, a PGE1 analog, for 8 weeks. The following assumptions were made for sample size calculation: (1) the wound-healing effect of a triple dose of rebamipide was equal to that of misoprostol; (2) the complete healing rates in the placebo and rebamipide groups were 20% and 58%, respectively; (3) 10% of patients in each group will either drop out or have unsatisfactory follow-up VCE images. We calculated that at least 30 patients were needed in the rebamipide group to give the study 90% power at a 5% significance level by a test of proportions.

**4. Ethics**

This study will be conducted in accordance with the Declaration of Helsinki, an ethical guideline for clinical studies.

**4.1 Consent of Subjects**

Before the study, the investigator, etc., will provide an adequate explanation on the contents of the study to the subject, on the basis of the informed consent form approved by the institutional review board of the study institution, and will obtain the signed written consent form from the subjects. The informed consent form will contain the contents specified in the standards stipulated in Article 14, Paragraph 3, and Article 80-2 of the Pharmaceutical Affairs Law (“Standards for the Implementation of Clinical Trials of Pharmaceutical Products” [GCP] [Ordinance No. 28 of the Ministry of Health and Welfare]).

**4.2 Institutional Review Board of the Study Institution**

Members of the institutional review board established by the head of the study institution will review the submitted protocol, informed consent form, etc. from the ethical, scientific, and medical standpoints; express their opinions in writing; and forward their report to the head of the study institution. The document containing their opinions will clearly specify the reviewed study, reviewed materials, date of review, and approval of the study. Deviations from the protocol or partial amendments to the protocol because of medically unavoidable reasons (e.g., to avoid risks to subjects) will be reported to and approved by the institutional review board.

**5. Monetary Payment and Medical Security**

In this clinical study, a partial self-pay burden will be applied for consultation and examinations, as in routine clinical practice; however, expenses for drugs used in the clinical study will not be borne by patients. In addition, there will be no special increase in expenses due to participation in the study.

If health hazards such as adverse reactions occur in patients during the clinical study, the physicians will perform appropriate consultation and treatment. Health insurance will be used. However, if health hazards occur in patients due to participation in the study, the treatment expenses will be paid by the hospital, and thus there will be no financial burden to the patients.

In conducting this clinical study, we have taken out the liability insurance offered by Tokyo Marine & Nichido Fire Insurance Co., Ltd. for clinical studies. If unpredictable serious health hazards occur and a causal relation to the study is revealed, compensation payment will be made depending on the degree of damage. The specific contents of compensation are shown in the Appendix.

**6. Changes in the Contents of the Protocol**

If changes to the protocol become inevitable after the initiation of the study, the study implementation manager will collaborate with the principal investigator and determine the contents to be changed. Changes can be made to an extent that is not deviating from the objective of the study.

**7. Study Period**

Case accumulation period: After approval to August 31, 2014

**8．Study Organization Meeting to Consider Drug-induced Small Intestinal Mucosal Damage**

**8.1 Study Manager**

Tetsuo Arakawa, Professor, Department of Gastroenterology, Osaka City University　Graduate School of Medicine

**8.2 Study Sites**

1. Department of Gastroenterology, Osaka City University Graduate School of Medicine, Osaka, Japan
2. Second Department of Internal Medicine, Osaka Medical College, Takatsuki, Osaka, Japan.
3. Department of Molecular Gastroenterology and Hepatology, Kyoto Prefectural University of Medicine, Kyoto, Japan.
4. Department of Internal Medicine and Gastroenterology, Saga Medical School, Saga, Japan

**9. References**

1. Patrono C. Am J Med. 110(1A):62S-65S, 2001.
2. [Pearson TA](http://www.ncbi.nlm.nih.gov/sites/entrez?Db=pubmed&Cmd=Search&Term="Pearson TA"%5BAuthor%5D&itool=EntrezSystem2.PEntrez.Pubmed.Pubmed_ResultsPanel.Pubmed_DiscoveryPanel.Pubmed_RVAbstractPlus) etal. 2002 update. Circulation 2002; 106: 388–91.
3. Cryer B et al. Gastroenterology 117:17-25,1999.
4. Derry S et al. BMJ 321:11:1183-1189,2000.
5. Joseph A et al. CMAJ 177:4:347-351,2007.
6. Sakamoto C et al. Eur. J. Pharmacol 62 765-772, 2006
7. Laine L et al. Aliment Pharmacol Ther 24:751-767,2006.
8. Wai K Leung et al. Lancet 369:614, 2007.
9. Sugimori S et al. Digestion 78:208-213, 2008
10. Lanas A et al. DDW 2008 AGA Institute Research Forum.
11. Watanabe T et al. Clinical Gastro. & Hepatol. 6:1279-1282, 2008.
12. Mizoguchi H et al. J Gastroenterol Hepatol 16:1112-1119,2001.
13. Arakawa T et al. Digestive Diseases and Science 50 :Suppl.Octo.2005.
14. Takemoto C et al　 The journal of adult diseases　19(4) 137-149, 1989 (Japanese).
15. Takemoto C et al　 The journal of adult diseases　19(4) 151-180, 1989(Japanese).
